# IoT-Enabled WBAN and Machine Learning for Speech Emotion Recognition in Patients

**DOI:** 10.3390/s23062948

**Published:** 2023-03-08

**Authors:** Damilola D. Olatinwo, Adnan Abu-Mahfouz, Gerhard Hancke, Hermanus Myburgh

**Affiliations:** 1 Department of Electrical, Electronic and Computer Engineering, University of Pretoria, Pretoria 0001, South Africa; 2 Council for Scientific and Industrial Research (CSIR), Pretoria 0184, South Africa; 3 Department of Computer Science, City University of Hong Kong, Hong Kong, China

**Keywords:** IoT WBAN, machine learning, deep learning, edge AI, speech emotion, CNN, BiLSTM, standard scaler, min–max scaler, robust scaler, data augmentation, spectrograms, regularization techniques, MFCC, Mel spectrogram

## Abstract

Internet of things (IoT)-enabled wireless body area network (WBAN) is an emerging technology that combines medical devices, wireless devices, and non-medical devices for healthcare management applications. Speech emotion recognition (SER) is an active research field in the healthcare domain and machine learning. It is a technique that can be used to automatically identify speakers’ emotions from their speech. However, the SER system, especially in the healthcare domain, is confronted with a few challenges. For example, low prediction accuracy, high computational complexity, delay in real-time prediction, and how to identify appropriate features from speech. Motivated by these research gaps, we proposed an emotion-aware IoT-enabled WBAN system within the healthcare framework where data processing and long-range data transmissions are performed by an edge AI system for real-time prediction of patients’ speech emotions as well as to capture the changes in emotions before and after treatment. Additionally, we investigated the effectiveness of different machine learning and deep learning algorithms in terms of performance classification, feature extraction methods, and normalization methods. We developed a hybrid deep learning model, i.e., convolutional neural network (CNN) and bidirectional long short-term memory (BiLSTM), and a regularized CNN model. We combined the models with different optimization strategies and regularization techniques to improve the prediction accuracy, reduce generalization error, and reduce the computational complexity of the neural networks in terms of their computational time, power, and space. Different experiments were performed to check the efficiency and effectiveness of the proposed machine learning and deep learning algorithms. The proposed models are compared with a related existing model for evaluation and validation using standard performance metrics such as prediction accuracy, precision, recall, F1 score, confusion matrix, and the differences between the actual and predicted values. The experimental results proved that one of the proposed models outperformed the existing model with an accuracy of about 98%.

## 1. Introduction

Due to the rapid advancement of the Internet of things (IoT) technology in various fields, ref. [1] IoT devices, IoT services, and IoT applications, IoT-enabled wireless body area network (WBAN) has gained popularity as well as new opportunities for its development [2,3,4]. In the IoT-enabled WBAN systems, IoT-connected devices, for example, audio recording devices and intelligent sensors, generate a huge amount of signals, such as multimodal health data. As a result of the big data generated by the IoT devices, effective data analysis, data processing, and prediction becomes very difficult. Consequently, incorporating machine learning methods with IoT-enabled WBAN becomes a necessity for effective analysis, processing and prediction of the health data. In this study, we proposed the integration of machine learning algorithms with IoT-enabled WBAN systems to predict patients’ emotions from their speech. The developed system would assist medical professionals to identify patients with mental health related issues such as depression, anxiety disorder, and bipolar disorder for early medication and therapy.

Speech can be described as an information-rich signal which contains linguistic and paralinguistic information. Emotion is one of the key instance of paralinguistic information which in part is conveyed by speech. As a consequence, it is important to develop machines that understand paralinguistic information, for example, emotion, to facilitate the monitoring of patients emotion from their speech. Emotions are mental states caused by neurophysiological shifts associated with different thoughts, feelings, behavioural responses, and a degree of pleasure or displeasure. The advent of IoT technology has made speech signals an excellent source to recognize the emotional state of humans either during human-to-human communication, human-to-machine communication or based on recorded human speech. At the same time, the emotional state of someone’s mind is also a critical factor in finding a correlation influencing channel of interactions between people, such as facial expressions, speech characteristics, and also the linguistic content of verbal communications [5,6]. In practice, emotions are economically more straightforward to obtain than other corporal signals like implantable data [7], electromyography (EMG) [8], and electroencephalogram (EEG) [9], which makes speech emotion recognition (SER) research very attractive.

SER research has recently been helpful in improving the health and well-being of humans, developing a healthy society or social behaviour, and addressing psychological issues to enhance humans’ productivity. Emotions have repercussions both on voice characteristics and the linguistic content of speech. Typically, a machine-learning algorithm is used to harvest language features with random mutations to categorize emotional responses into different class. The features that one may pick to train the machine learning model is one of the most important tools for developing effective SER systems that recognizes a patient’s emotions. To understand the relevance of the raw signal in the speech data, it is essential to increase the diversity of the conversation and response appropriately [10,11,12].

The features extracted from the speech signal greatly influence the reliability of human–computer interaction. The speech signals have discrete feature representations that suffer from data sparsity and do not consider semantic relatedness between features to enhance the personification of machines or improve recognition efficiency when converting speech into a feature representation that can represent human emotions. Thus, selecting an optimal set of features that is less time consuming, efficient and generalized with unseen raw signals becomes a challenge because it is quite challenging to find the features that perfectly represent each emotional state  [12,13,14,15,16]. In literature, the prediction accuracy as well as the computational complexity of the existing SER system still requires more improvement, especially in the healthcare domain, to achieve a viable SER solution. Another challenge in SER is the ability to capture the changes in the emotion of patients in a real-time manner before and after treatment. As a consequence, we propose an emotion-aware IoT-enabled WBAN system within the healthcare framework by investigating the effectiveness of different machine learning and deep learning algorithms, feature extraction methods, and normalization methods. Additionally, we propose a hybrid deep learning model combined with different optimization strategies to improve the prediction accuracy as well as to minimize the computational complexity of the SER system in terms of computational time, power, and space. The main contributions of this study are summarized as follows:

- The integration of machine learning methods with IoT-enabled WBAN systems for patients’ speech emotion detection;
- The development of an emotion-aware IoT-enabled WBAN system with edge AI for real-time predictions and to capture the changes in patients’ emotions before and after treatment;
- The combination of signal processing techniques and deep learning methods for extracting speech emotion features;
- The development of a hybrid deep learning model, i.e., convolutional neural network (CNN) and bidirectional long short-term memory (BiLSTM), and a regularized CNN model combined with different optimization strategies and regularization techniques to improve the prediction accuracy as well as to minimize the computational complexity of the SER system in terms of computational time, power, and space;
- The effect of feature normalization methods and their impact on the classification performance of the SER models were investigated;
- The comparison and evaluation of the proposed SER models with a related existing model for the sake of validation were carried out.

The content of this paper is organized as follows, Section 1 presents the introduction, including motivation, objective, and the contribution summary of the study. Section 2 discusses a short literature review of the machine and deep learning-related studies for SER in chronological order. Section 3 describes the proposed methods. Section 4 discusses the performance metrics used for evaluating the models. Section 5 presents the experimental results and discussions, while Section 6 concludes the work.

## 2. Related Works

For a long time, SER research has been thrilling and several research papers have provided various methods for emotion recognition from speech expressions, body gesture, and audio-visual expressions [17,18,19]. In SER, one of the central research issues is how to mine non-discriminating relevant features from speech signals to improve information diversity. In recent years, several researchers in the health domain have proposed different methods for extracting features to construct emotion recognition systems, for example, ref. [20] developed an emotion detection system towards IoT big data from patients’ speech using features with four multiple directions and from video using a local ternary pattern. The authors of [21] proposed a systematic and qualitative analysis of EEG features using multiple feature selection methods. It was concluded that multivariate feature selection techniques outperformed the univariate techniques, for instance, advanced feature selection methods such as higher-order crossings, higher-order spectra, and Hilbert–Huang spectrum were compared with spectral power bands. Additionally, ref. [22] employed machine learning methods such as support vector machine (SVM) and artificial neural networks to develop emotion recognition systems using EEG signals of 15 participants. A wavelet analysis feature extraction technique was employed and features such as Hjorth parameters, statistical features, symmetric electrodes, differential entropy were extracted.

Likewise, different methods have been proposed in the literature for detecting relevant features directly from raw audio samples to recognize human emotions. In this direction, ref. [10] presented characteristics derived from prosody, spectral envelope, and voice quality, as well as their capability to distinguish emotions from speech. They employed 324 spectral and 54 prosody features integrated with five voice quality features to test their proposed method on the Surrey Audio-Visual Expressed Emotion (SAVEE) database [23] after involving minimal redundancy maximal relevance (mRMR) to reduce less discriminating features.

The authors of [24] utilized SVM to classify male speech samples from the RAVDESS dataset. They used a continuous wavelet transform (CWT) when choosing features and fed them to different SVM classifiers. The authors of [25] presented a multimodal emotion recognition technology that employs both the speech and facial data to recognize emotions in RAVDESS datasets. A pre-trained CNN-14 as a transfer model stack on Bi-LSTM with attention was used to distinguish acoustic features.

In [26,27,28,29], authors used a CNN model to understand the thoughts and emotions in people’s conversations by extracting the audio file and stacking the generated matrix in a one-dimensional cluster and summing the mean values along the time axis. However, the raw signal information quality is degraded and not efficient for large corpora.

In [30], authors presented a majority voting technique (MVT) for detecting speech emotion using a fast correlation-based feature (FCBF) and Fisher score algorithms for feature selection. They extracted 16 low-level features and different experiments were performed using several ML algorithms, including neural network (NN) with one hidden layer, classification and regression tree (CART), SVM and K-nearest neighbour (KNN) on the Berlin Emotion Speech Database (EMO-DB) [31].

Authors of [32] used Mel Frequency Cepstral coefficients (MFCCs) produced from 520 samples taken from the EMO-DB dataset to develop a speech emotion detection system. The framework was implemented using a feature reduction fuzzy C-means clustering. They employed several classifiers such as NN, SVM and KNN. Additionally,  ref. [33] used the MFCC, chromatogram, zero-crossing rate, log mel-scale spectrogram, and the statistical root means square to extract emotion feature representation from relevant signals in audio files. The feature representations were used as inputs into the CNN model to outline local features stack on both LSTM and GRU architectures so as to understand the long-term contextual sequence in a sentence.

The exploration of acoustic features, semantic primitives, and emotion dimensions to map raw audio files into emotional expression was considered in [15]. They categorized the continuous emotion dimensional importance into basic classes using the logistic model trees for multilingual speech emotion recognition on Fujitsu, EMO-DB [31], and SAVEE databases. The highest weighted average precision was obtained after performing speaker normalization and feature selection.

In [34], the authors used the channel attention module in a multilayer perceptron (MLP) model to extract the channel features or locations in the RAVDESS [35], EMO-DB, and IEMOCAP speech emotion spectrograms. They used spatial attention in the CNN to extract the spatial features from the training dataset. The channel and spatial features were merged as a self-attention module and used CNN as a classifier.

The author of [36] proposed a speech emotion detection system for healthcare using the RAVDESS dataset. Audio IoT devices are employed to record human voices and predict their emotions through deep learning, i.e., 2D-CNN. Furthermore, the study employed data normalization and augmentation techniques to improve the performance of the system. It is important to emphasize that emotion recognition is still a tremendous challenge for several reasons, as mentioned in Section 1. These reasons include the existence of a gap between acoustic feature selection and human emotions, and the non-existence of a solid theoretical foundation relating the characteristics of voice to a human’s emotions [11]. Therefore, more studies are still required to investigate the design of new models.

Different from the above papers, we proposed the integration of machine learning methods with IoT-enabled WBAN systems to identify patients’ emotion from their speech. In this system, data transmission and data processing are performed using edge AI for real-time prediction and as well capture the changes in patients’ emotions before and after treatment. Also, we proposed two deep learning models—the first model is a hybrid CNN and BiLSTM combined with different optimization strategies and the second model is a regularized CNN model using dropout, L1, and L2 techniques. The hybrid deep learning and the regularized CNN models are constructed to improve the prediction accuracy as well as to minimize the computational complexity of the SER system in terms of computational time, power, and space. Additionally, since speech data are generally composed of different emotions, we used the MFFCs signal processing techniques to extract feature vectors from the speech emotion signals. We applied different feature normalization methods to investigate their impacts on the classification performance.

## 3. Proposed Method

This section presents the data collection process, exploratory data analysis, data processing, architecture of the proposed system, channel model, and model building process in the following subsections.

### 3.1. Data Collection

To achieve the goal of improving the health and well-being of patients with emotional issues, similar to other related studies on SER in healthcare, for example, ref. [36], we considered the RAVDESS dataset which is a standard state-of-the-art speech dataset published in [35]. The dataset includes all the features we are interested in to identify or predict emotions in our simulation study and experiment. This will provide an insight into the implementation of the work in a real-life scenario. We employed the dataset so that the developed system could generalize well, i.e., the ability for the developed system to adapt properly to new or unseen data. We split the dataset into 80% for training (i.e., 1296 samples), 10% for validation (i.e., 72 samples), and 10% for testing (i.e., 72 samples). The following subsections provide an overview of the dataset based on the 5 V’s of Big Data.

#### 3.1.1. Volume

Volume is used to quantify the amount and size of the data points in the dataset. The speech dataset has a total of 1440 files with a size of 24.8 GB.

#### 3.1.2. Variety

Variety is used to refer to the different types of data found in the dataset which include the raw data, unstructured data, and semi-structured data. The speech dataset is a multipurpose dataset that has the audio-visual, audio-only, and video-only modalities. Additionally, it is important to mention that each of the 1440 files contains a unique filename which has a 7-part numerical identifier (i.e., 03-01-06-01-02-01-12.wav). The identifiers define the stimulus characteristics. The dataset has 24 professional actors, i.e., 12 females and 12 males. Each of the actors utters 60 different intonations of speech with emotions. The filename identifiers are modality (01 = full-AV, 02 = video-only, 03 = audio-only), vocal channel (01 = speech, 02 = song), emotion (01 = neutral, 02 = calm, 03 = happy, 04 = sad, 05 = angry, 06 = fearful, 07 = disgust, 08 = surprised), emotional intensity (01 = normal, 02 = strong), statement (01 = “kids are talking by the door”, 02 = “dogs are sitting by the door”), repetition (01 = 1st repetition, 02 = 2nd repetition), and actor (from 01 to 24, where the male actors are denoted with odd numbers and female actors are denoted with even numbers). Therefore, the  dataset has a lot of variety and is suitable for this research work.

#### 3.1.3. Veracity

Veracity is used to measure the truthfulness or accuracy of data. For the purpose of emotion validity, each recording was rated 10 times. Additionally, the  dataset has a proportional number of files for each emotion, and this prevents the problem derived from training algorithms with un-balanced data. In addition, the dataset is a well-referenced dataset in the research community and has being used by several authors, for instance [13,15,17,25]. As a consequence, we are confident about the veracity of the  dataset.

#### 3.1.4. Velocity

Velocity is the speed at which the data are received, stored, and managed. The dataset has a high velocity and this makes it a valid dataset for this project.

#### 3.1.5. Value

The dataset brings a significant value to the research community, industry, and health domain. The outcome of this research work can be used to predict any speaker’s speech emotion. For instance, it can be used in hospitals to enhance the therapy section of patients. It can also be used to identify stressed and depressed patients. Therefore, there is a lot of value provided by the dataset and that is why it was chosen for this project.

### 3.2. Exploratory Data Analysis (EDA)

**Figure 1:**
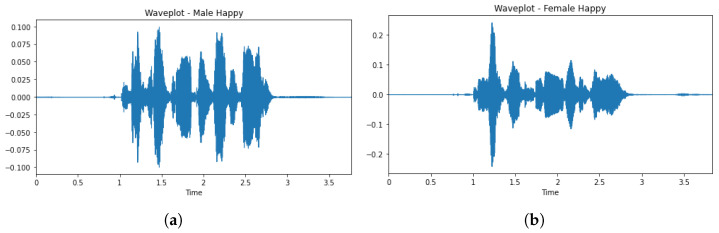
(**a**) Raw male speech signal. (**b**) Raw female speech signal.

**Figure 2:**
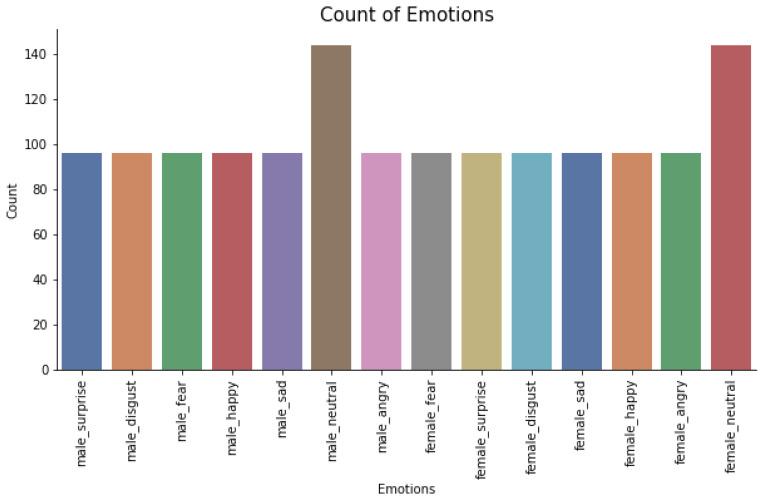
Distribution of labels (emotions).

The EDA phase was used to study the dataset to know more as well as to understand the dataset better by visualizing the raw audio signals as shown in Figure 1a,b. This was achieved by visualizing the distribution of classes (emotions) in the dataset to check whether the dataset is balanced as shown in Figure 2. From Figure 2, it was observed that the dataset is good.

### 3.3. Proposed IoT-Enabled WBAN SER Framework with Edge AI

Generally, being healthy is multifaceted and for optimal wellness to be achieved, it is not enough to obtain only one form of health [37]. As a consequence, other forms of wellness such as emotional wellness, mental wellness, and physical wellness must be considered. It is important to mention that human happiness as well as mental health have a close link with being emotionally well. Recently, research has proven that emotional stress could affect human physical wellness. Consequently, emotional wellness contributes to human productivity and helps to realize one’s full potential [38]. In this paper, we propose a novel SER framework for an IoT-enabled WBAN system to assist medical professionals to improve the health and well-being of patients suffering from depression. The proposed framework consist of a patient, an audio recording device, an access gateway, an edge AI technology, a cloud system, and a remote medical centre. Following this, the speech data are generated by attaching an IoT audio recording device to a patient’s body towards the mouth region.

#### 3.3.1. IoT Audio Device

The audio recording device consists of four key modules integrated with a Raspberry Pi. The modules are a MAX9814 electret microphone [39], microprocessor, memory, and a transceiver. The electret microphone is an electrostatic capacitor-based microphone that does not require a polarizing power supply resource because it uses a permanently charged material. Similar to other related studies, the MAX9814 electret microphone is considered in this work for collecting the speech data because it is able to record sounds with amplification [36]. The microprocessor module is responsible for converting the analogue speech data into digital form. The memory module is used for storing the digital speech data. Whereas, the transceiver is in charge of the data transmission and reception. However, because the generated digital speech data are usually very large and cannot be processed by the devices, then the device transceiver forwards the digital speech data through a short-range communication technology to an access gateway.

#### 3.3.2. Access Gateway

**Figure 3:**
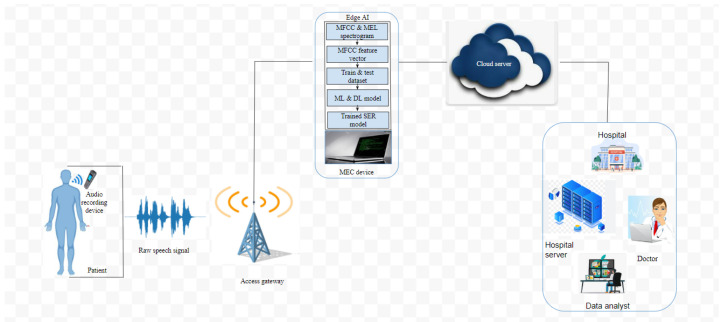
IoT-enabled WBAN SER framework.

As shown in Figure 3, the access gateway acts as an intermediary between the IoT audio device and the edge AI technology. It coordinates and allocates channel, time, and power resources to the devices [40]. It obtains the digital speech data from the audio device and forwards it to an edge AI system.

#### 3.3.3. Edge AI System

An edge AI System is a computing paradigm that allows computation outside the cloud, i.e., at the network’s edge, especially in applications such as WBAN where long-range data transmission and data processing are burdens on the network due to its limited resources. To improve the efficiency of the IoT-enabled SER system in terms of real-time data transmission and data processing, we employ a mobile edge computing (MEC) device. The MEC device processes the raw speech data and uses the developed SER algorithm to predict patients’ emotions [41]. Therefore, the outcome of the prediction is made available in real time at the patient’s site and also at the doctor’s office, and this helps to capture the changes in patients’ emotions before and after treatment.

Unlike the conventional cloud-based SER architecture, the edge-based SER architecture has different advantages including low delay, low energy consumption, and high quality of service. Thus, integrating an edge AI-based system with the IoT-enabled WBAN SER system would help to achieve a real-time prediction of patients emotion as well as capture the changes in the patients’ emotions before and after treatment.

#### 3.3.4. Cloud Server

The cloud server could as well be employed for data processing and predictions, but for the case of the proposed IoT WBAN SER, the data processing and predictions are performed at the network’s edge because of its numerous advantages such as closeness to the users/devices, low delay, low energy consumption, and high quality of service. Additionally, if the huge speech data generated by the audio recorder are forwarded to the cloud, this will result in unpredictable delays and would not be able to provide timely predictions due to the return delay of the remote cloud server [42]. Thus, for efficiency, the MEC forwards the predicted emotions to the cloud server and from the cloud server to a remote medical server.

### 3.4. Channel Model for Data Communication in WBAN

A channel model is considered in this study to mathematically represent the effects of the communication channel used to propagate the collected speech data from a patient to an access point device. Based on the IEEE 802.15.6 standard, WBAN has three types of communication channels, namely on-body, in-body, and off-body channels. For the purpose of this work, we consider the on-body communication channel. As a consequence, during the modelling of a WBAN communication channel, different channel characteristics such as path loss, fading, shadowing, and power decay are considered. Therefore, for the WBAN environment, we consider the effect of path loss, shadowing, fading, and power decay. The path loss of the communication channel assumes a log-distance path loss model with a path loss exponent of the considered communication environment. Additionally, a small-scale fading effect is considered using a Rayleigh fading model. Following this, the path loss between an audio device and the access gateway is modelled based on the empirical power decay law [43,44] in (1) as:

(1) $$P_{d}=P_{d_{0}}+10\;n\log_{10}\frac{d}{d_{0}}$$

In (1), $P_{d}$ denotes the path loss in dB, $P_{d_{0}}$ is a reference distance, and the path loss exponent is denoted as *n*. Moreover, the human body movement varies based on the environment and this introduces shadowing effect in the communication channel. Thus, integrating shadowing gives a total path loss [45] expressed in (2) as:

(2) $$P=P_{d}+S$$

where S denotes the shadowing factor.

### 3.5. Data Processing

This phase was used for converting the speech signal from its original form to a much more usable form. For this to be achieved, various methods such as data argumentation, feature extraction, feature normalization, and addressing categorical data were considered and are discussed in details in the following subsections.

#### 3.5.1. Data Augmentation

The data augmentation technique was used in this project to create new polymerized data samples by adding small disturbance to the initial training set. To generate a polymerized data for the speech data, noise injection, shifting time, changing pitch, and speed are applied. This idea is to enable the developed model to be invariant to disturbance and also enhance its ability to generalize well.

#### 3.5.2. Feature Extraction

One of the most crucial aspects of SER is feature extraction, as this helps to extract unique discriminative features from a speaker’s speech. The features could then be used to identify the emotions of human beings. In other words, feature extraction is used to generate the feature vectors that represents each speech data point. Since the speech data consist of a mixture of different emotions, to distinguish the speech emotion signals, we extracted the feature vectors of the speech emotion signals using the MFCCs signal processing technique [46]. MFCCs is the result obtained from taking the inverse of the Discrete Fourier Transform (DFT) applied on a logarithmic spectrum. MFCCs can be determined by employing a psychoacoustically motivated filter-bank, a logarithmic compression, as well as a discrete cosine transform. Let us assume the output of an N-channel filter-bank is *Y*(*n*), *n* = 1, 2, …, *N*, then the MFCC is modelled in (3) as:

(3) $$\zeta_{m}=\sum_{n=1}^{N}\;\left[\log\;Y(n)\right]\;cos\left[\frac{\pi m}{N}\left(n-\frac{1}{2}\right)\right]$$

**Figure 4:**
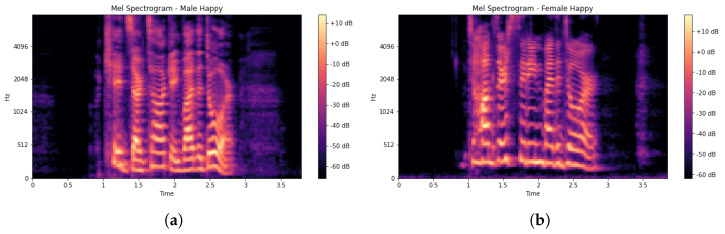
(**a**) Male spectrogram representation. (**b**) Female spectrogram representation.

In (3), *m* denotes a cepstral coefficient index. The Mel spectrogram, which is a Fast Fourier Transform (FFT) technique, was computed on the overlapping windowed segments of the SER signal. Usually, a spectrogram (*S*) is a visual way of representing signal strength and is used to display frequency sound waves as shown in Figure 4a,b. The MFCC feature vector generation scheme is presented in Algorithm 1. A spectrogram is a combination of frequencies in relation to time. The x-axis denotes time (*T*) while the y-axis denotes the frequency (*F*) range of each input signal (*X*) and is modelled in (4) [47] as:

(4) $$X\left(\tau ,\omega \right)=\int_{-\infty }^{\infty }S\left(T\right)\;F\left(T-\tau \right)\;exp{}^{i}{}^{\omega }{}^{T}\;dt$$

**Algorithm 1:** Feature Vector Generation Scheme
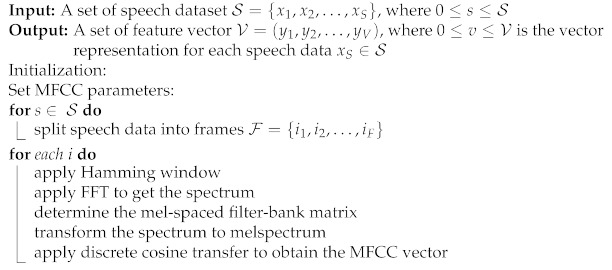


#### 3.5.3. Feature Normalization

After the features were extracted from the speech, we applied different normalization methods such as the Z-score, min–max, and robust scaler methods. The purpose of the normalization method is to enhance the accuracy of the feature by maintaining the affine invariance of the signal. The following subsections present a brief discussion on the normalization methods.

(i) Z-score or Standard Scaler

The Z-score normalization method was employed from scikit-learn and applied to the extracted speech features. This normalization technique was used to remove the mean and scale the data to a unit variance. Given a speech feature *P* and the original value *x*, the mean (μ) and the standard deviation (σ) of the speech features were first calculated and then the standardized feature (*Z*) was modeled in (5) [48] as:

(5) $$Z=\frac{x-\mu (P)}{\sigma (P)}$$

(ii) Min–max Scaler

We used the min–max scaler from scikit-learn to transform each speech feature by scaling them individually to a given range on the training set and is modeled in (6) as:

(6) $$V=\frac{x-\min(x)}{\max(x)-\min(x)}$$

where *V* is used to denote the normalized value.

(iii) Robust Scaler

The robust scaler was applied to remove the median as well as scale the data based on the quantile range. This scaler scales the speech features using robust statistics, such as interquartile range, to the outliers. The robust scaler is modelled in (7) as:

(7) $$RS_{\left(P\right)}=\frac{P-Q_{1}\left(x\right)}{Q_{3}\left(x\right)-Q_{1}\left(x\right)}$$

where $RS_{P}$ is the normalization value, $Q_{1}$ and $Q_{3}$ denote the 1st interquartile and the 3rd interquartile, respectively.

#### 3.5.4. Addressing Categorical Data

In this study, we employed the one hot encoding technique to address the data that are categorical in nature. Basically, the one hot encoding technique is used for converting categorical data variables that needs be fed into the machine learning or deep learning algorithms into numerical data variables to improve the prediction and classification accuracy of the model. Specifically, the one hot encoding technique was used to convert the label *Y* from a categorical data (non numerical label) to a numerical data (numerical label).

### 3.6. Model Building

**Figure 5:**
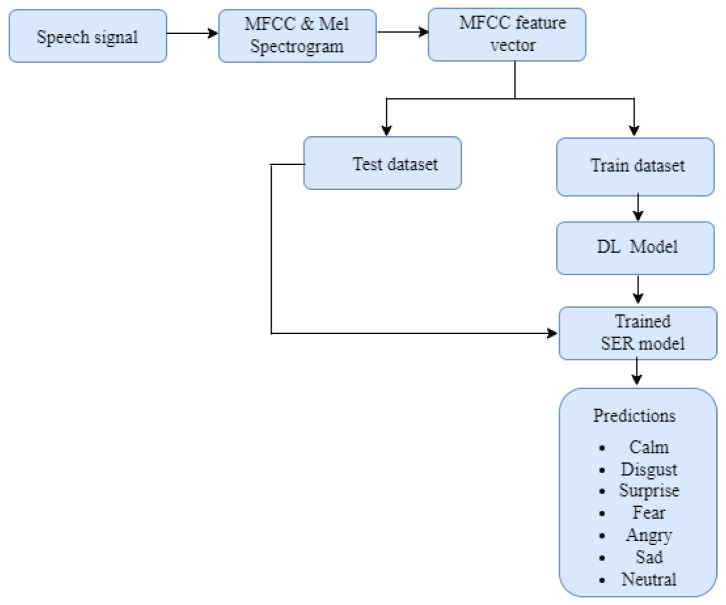
Proposed system architecture.

The model building process follows the architecture in Figure 5. The architecture consists of two major phases including the training phase and the prediction phase. During the training phase, the speech signals are fed into the system as input, then the speech signal spectrogram representations and the feature vectors are extracted. Afterwards, the labelled feature vectors are fed into the developed machine learning and deep learning models to learn the feature vector representations. During the prediction phase, the SER signals are fed into the trained model and the model predicts the eight classes of emotions, (i.e., calm, disgust, surprise, fear, angry, sad, neutral, and happy). Scikit-Learn and TensorFlow were employed to train the machine learning algorithms. Random search optimization method was used to find the optimal hyperparameters. We discuss the parameters of the machine learning and deep learning algorithms in the subsections below.

#### 3.6.1. K-Means and Principal Component Analysis (PCA)

**Figure 6:**
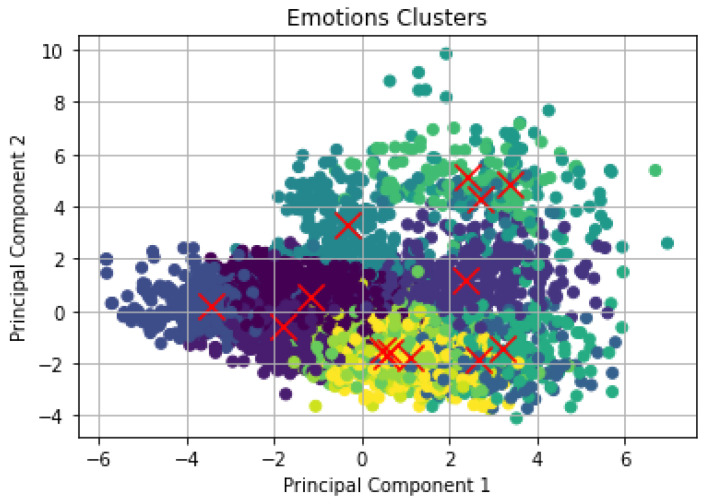
Clustering of speech data using k-means and PCA.

To investigate the number of emotions that can be extracted from the speech dataset, an unsupervised learning approach was applied to group the emotion classes in the dataset into clusters. We employed the k-means clustering algorithm to cluster the data and the clustered speech emotion is presented in Figure 6. It was also used for making an inference from the dataset by using only the input vectors. The goal of k-means is to group together similar data points. In addition, the k-means algorithm was used to discover the hidden patterns within the dataset. For this to be achieved, k-means searches for a fixed k number of clusters in the dataset, and k was set to 14 as the number of emotions. Additionally, PCA was applied for the dimensionality reduction of the data by converting a large set of variables to a small set of variables without losing most of the information contained in the large set. The result of the clustered data using k-means and PCA is presented in Figure 6.

#### 3.6.2. K-Nearest Neighbors (KNN)

The KNN classifier belongs to the instance-based family, also referred to as lazy learning algorithms [11]. It works by taking a data point and finding the k nearest labelled data points. The KNN classifier was trained with four nearest neighbors, i.e., k = 4 and this gives the best accuracy results.

#### 3.6.3. Decision Tree (DT)

This is a class of machine learning algorithms capable of performing multiclass classification on a dataset. The decision tree algorithm was employed in this study to build a decision tree model for the dataset.

#### 3.6.4. Convolutional Neural Networks (CNN) and Bidirectional Long Short-Term Memory (BiLSTM)

This section presents a hybrid convolutional bidirectional long short-term memory deep learning model. The model consists of three one-dimensional convolutional layers with a rectified linear unit (ReLU) activation, a max pooling layer, and a batch normalization layer each. The three convolutional layers are stacked on each other. The batch normalization layer is employed to make the neural network train faster and more stable by normalizing the input layer through re-scaling and re-centering. The first convolutional layer has 2048 units, the second convolutional layer has 1024 units, and the third has 512 units. The output from the third convolutional layer was fed into a BiLSTM layer. The BiLSTM layer consists of two LSTMs, namely, a forward LSTM and a backward LSTM. The forward LSTM was used to learn the input feature vector in a forward direction and in a reverse direction through the backward LSTM. The use of the BiLSTM network helped to capture the contextual information within the vector representations. The outputs of the two LSTMs were combined to obtain a vector representation of the speech. Following this, the output of the BiLSTM layer was fed to a dense layer with a softmax activation fuction. The softmax activation function uses the input feature vector to predict emotions. This is expressed in (8) as:

(8) $$S\left({\vec{p}}_{i}\right)=\frac{e^{p_{i}}}{\sum_{j=1}^{K}\;e^{p_{j}}}$$

In (8), softmax is denoted as *S*, ${\vec{p}}_{i}$ is the input vector, and *K* is the number of emotion classes. Whereas, $e^{p_{i}}$ and $e^{p_{j}}$ represents the standard exponential function of the input and output vectors. The model has two major parts, namely, the feature learning (extraction part) and the FCL (classification part). The feature extraction part of the model uses the convolutional layers to extract the patterns of the speech emotion from the feature vector representations of the speech emotion signals. The extracted features are then passed to the FCL for detection and classification of different emotions using the softmax function.

#### 3.6.5. Regularized Convolutional Neural Networks (RCNN)

In this section, we present the development of a CNN model that is composed of a four one-dimensional convolutional layers that are stacked on each other. The first two convolutional layers have 256 units, the third convolutional layer has 128 units, and the fourth convolutional layer has 64 units. Each of the layers uses a ReLU activation function. Each convolutional layer is preceded by a MaxPooling layer. Dropout, L1, and L2 techniques were used for regularization at the first layer. The regularization technique was employed to reduce the computational complexity of the network and to also increase the prediction accuracy of the system. Furthermore, a softmax activation function was applied at the fully connected layer (FCL) to predict emotions.

## 4. Evaluation

The performance of the proposed models was evaluated by using performance evaluation metrics that included classification report (prediction accuracy, precision, recall, and F1 score) and confusion matrix. The performance parameters are discussed next.

### 4.1. Prediction Accuracy

The prediction accuracy $\left(P_{A}\right)$ is an example of a classification report used to determine the percentage of the correctly predicted samples from the given samples and is expressed in (9) as:

(9) $$P_{A}=\frac{T_{p}+T_{n}}{T_{p}+T_{n}+F_{p}+F_{n}}$$

### 4.2. Recall

Recall (ℜ) is used to measure how efficient the model is at predicting the number of positive samples from the given positive samples in the dataset and is expressed in (10) as:

(10) $$ℜ=\frac{T_{p}}{T_{p}+F_{p}}$$

### 4.3. Precision

The precision metric denoted as *℘* is used to determine the total number of relevant predicted positive samples and is expressed in (11) as:

(11) $$℘=\frac{T_{p}}{T_{p}+F_{p}}$$

### 4.4. F1-Score

The F1-score measure denoted as $F_{s}$ is determined by the harmonic mean of recall and precision and is expressed in (12) as:

(12) $$F_{s}=2\left[\frac{℘*ℜ}{℘+ℜ}\right]$$

In (7)–(11), $T_{p},T_{n},F_{p}$, and $F_{p}$ denote true positive, true negative, false positive, and false negative, respectively.

### 4.5. Confusion Matrix

Confusion matrix was used as a performance evaluation tool to summarize the performance of the classification model. It helps to understand the performance of the classification model and the type of errors that a model produced. It also provides a summary of the correct and incorrect predictions, while evaluating the performance of a classification model, under the followinf four categories:

#### 4.5.1. True Positives (TP)

True positives occur when the predicted observation belongs to a certain class and the observation actually belongs to that class.

#### 4.5.2. True Negatives (TN)

True negatives occur when the predicted observation does not belong to a certain class and the observation actually does not belong to that class.

#### 4.5.3. False Positives (FP)

False positives occur when the predicted observation belongs to a certain class but the observation actually does not belong to that class. FP error is called a Type I error.

#### 4.5.4. False Negatives (FN)

False negatives occur when the predicted observation does not belong to a certain class but the observation actually belongs to that class. FN error is called a Type II error.

## 5. Results and Discussion

In this section, we present the experimental results of the proposed SER systems. The performance of the developed machine learning and deep learning approaches, such as KNN, DT, and CNN, was compared based on the considered normalization methods using standard performance metrics such as accuracy, precision, recall, F1-score, and confusion matrix.

### 5.1. Experiment Settings

In the proposed system, we employed Adam optimization, random search hyperparameter optimization, and regularization techniques to optimize the computational resources such as computational time, computational power, and computational space of the system.

The parameters used for the experiments are shown in Table 1. The proposed models were tested on 10% of the data and the classification reports, i.e., accuracy, precision, recall, and F1-score, of the models are presented in Table 2, Table 3 and Table 4. The proposed deep learning models are compared with the most relevant existing work in the literature for the purpose of evaluation and validation. These reports were averaged based on the weights of each class.

### 5.2. Performance Comparison of the Proposed Models and the Existing Model

**Figure 7:**
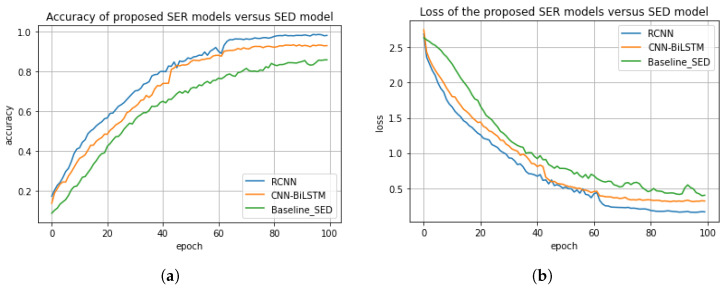
(**a**) Accuracy comparison of the proposed system versus baseline system. (**b**) Loss comparison of the proposed system versus baseline system.

This section presents the performance comparison of the proposed models, i.e., regularized CNN and CNN-BiLSTM and the most closely related existing model [36] in the context of accuracy, precision, recall, and F1 score, as shown in Table 2. In this experiment, the proposed regularized CNN model achieved an accuracy score of about 98%, a precision score of 95%, a recall score of 93%, and an F1 score of 92%. Whereas, the proposed CNN-BiLSTM model achieved an accuracy score of 85%, a precision score of 82%, a recall score of 80%, and a F1 score of 81%. The baseline model [36] achieved an accuracy score of 95%, a precision score of 92%, a recall score of 92%, and a F1 score of 90%. The accuracy and the loss plots of the models are shown in Figure 7a,b. We also compared the three models based on the difference between their actual values and their predicted values. For the proposed models, i.e., regularized CNN and CNN-BiLSTM, the difference between the actual value and the predicted value are about 10.5% and 22%, respectively. The difference between the actual value and the predicted value for the baseline model is about 10.5%. As a consequence, the regularized CNN outperformed both the CNN-BiLSTM and the existing model with a significant improvement of about 13.2% and 3%, respectively. This significant improvement could be attributed to the regularized techniques, i.e., L1, L2, and dropout, we employed. This is due to the fact that the regularized techniques were able to minimize computational complexity, minimize generalization error, and enhance the performance accuracy of the model.

### 5.3. Impact of Standard Scaler on the Performance of the Models

**Figure 8:**
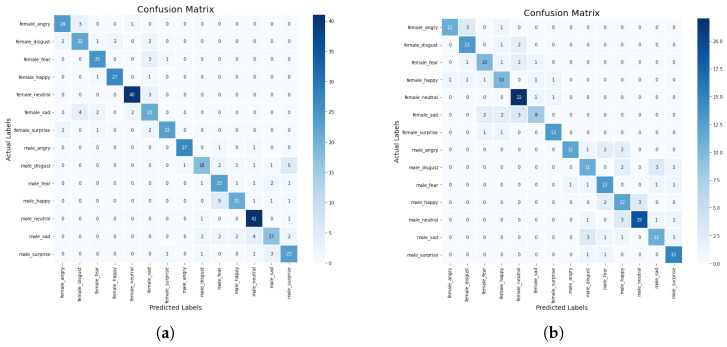
(**a**) Confusion matrix for CNN-BiLSTM model with standard scaler, (**b**) Confusion matrix for regularized CNN model with standard scaler.

**Figure 9:**
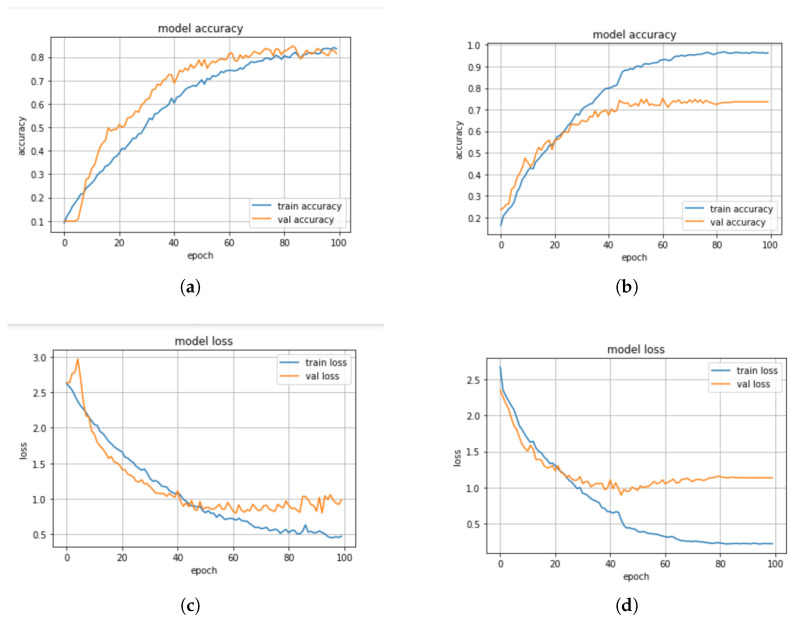
(**a**) Accuracy plot for the CNN-BiLSTM model with standard scaler, (**b**) Accuracy plot for the regularized CNN model with standard scaler, (**c**) Loss plot for the CNN-BiLSTM model with standard scaler, (**d**) Loss plot for the regularized CNN model with standard scaler.

In this section, we discuss some experiments conducted to compare the performance of the machine learning and deep learning models based on the standard scaler method. The confusion matrix of the CNN-BiLSTM and the regularized CNN models, which are the best models, are presented in Figure 8a,b, respectively, while their accuracy and loss plots are shown in Figure 9a–d, respectively.

From the results presented in Table 3, it can be observed that the KNN model achieved an accuracy score of 52%, a precision score of 91%, a recall score of 52%, and an F1-score of 64%, while the DT model obtained an accuracy score of 45%, a precision score of 42%, a recall score of 41%, and an F1-score of 41%. The CNN-BiLSTM model achieved an accuracy score of 85%, a precision score of 82%, a recall score of 80%, and an F1-score of 81%. In this experiment, the regularized CNN model performed better than the other models as it achieved an accuracy score of 98%, a precision score of 95%, a recall score of 93%, and an F1-score of 92%.

In addition, we compared the actual value and the predicted value of the CNN-BiLSTM and the regularized CNN models to investigate their performance. For the CNN-BiLSTM model, the difference between the actual value and the predicted value is about 22% while for the regularized CNN model, it is about 10.5%. Thus, it was observed that the two models performed well when the standard scaler method was applied. However, the regularized CNN performed better with a significant improvement of about 13.3%. The significant performance could be attributed to the optimization strategies that were employed to optimize the network parameters in order to find the optimum parameter. Moreover, different regularization techniques such as dropout, L1, and L2 are employed and this helped to reduce the computational complexity of the model in terms of computational time, power, and space.

### 5.4. Impact of Min–Max Scaler on the Performance of the Models

**Figure 10:**
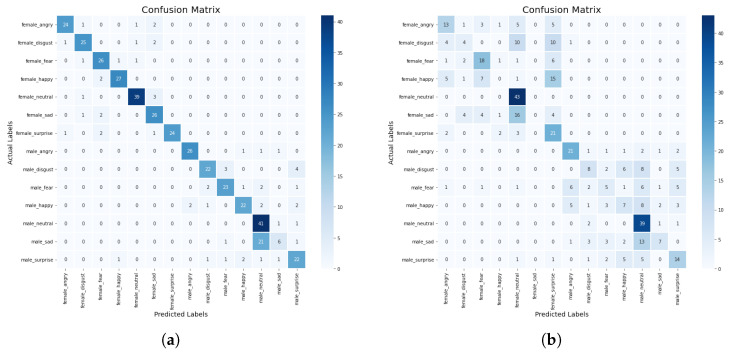
(**a**) Confusion matrix for CNN-BiLSTM model with min–max scaler. (**b**) Confusion matrix for regularized CNN model with min–max scaler.

**Figure 11:**
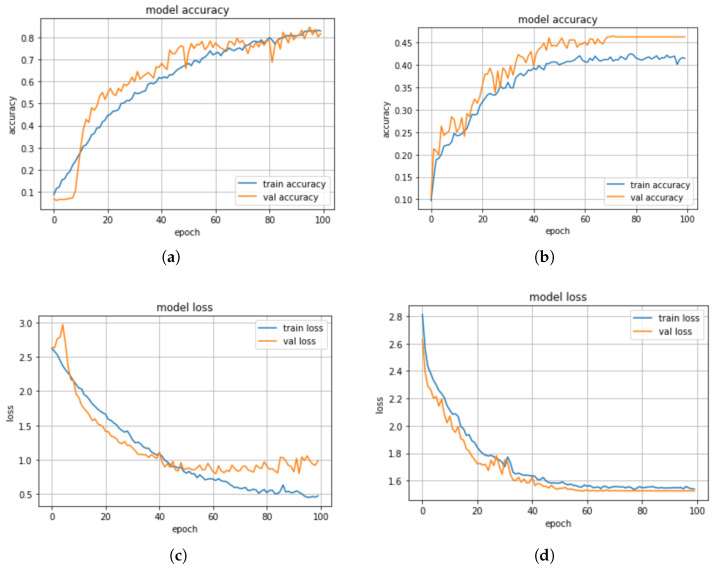
(**a**) Accuracy plot for the CNN-BiLSTM model with min–max scaler, (**b**) Accuracy plot for the regularized CNN model with min–max scaler. (**c**) Loss plot for the CNN-BiLSTM model with min–max scaler. (**d**) Loss plot for the regularized CNN model with min–max scaler.

When the min–max scaler method was applied on the models, we observed from the results in Table 4 that the KNN model has a higher precision score of 93% compared with DT with a precision score of 40%. Whereas, the CNN-BiLSTM achieved a 80% precision score, and the regularized CNN has a precision score of 40%. The CNN-BiLSTM outperformed the other models with an accuracy of 83%, a recall of 81%, and an F1-score of 80%. The regularized CNN model achieved an accuracy score of 47%, a recall score of 68%, an F1-score of 68%, while the KNN model achieved an accuracy score of 53%, a recall score of 52%, a F1-score of 65%, the DT model achieved an accuracy score of 44%, a recall score of 42%, an F1-score of 41%. The CNN-BiLSTM model performed better than the machine learning models and the regularized CNN model. The confusion matrix of the CNN-BiLSTM and the regularized CNN models are presented in Figure 10a,b. The accuracy and loss plots for the CNN-BiLSTM and regularized CNN models are shown in Figure 11a–d, respectively.

Additionally, the actual and the predicted values of the CNN-BiLSTM and regularized CNN models were compared. We observed that the difference between the actual and predicted values for the CNN-BiLSTM model is about 10.5%, while the difference between the actual and predicted values for the regularized CNN model is about 50%. In this experiment, the CNN-BiLSTM has the highest performance in terms of accuracy, precision, recall, and F1 score. The CNN-BiLSTM has a significant improvement of about 43% over the regularized CNN. The significant improvement is a result of the different optimization strategies that were employed to optimize the network parameters to find an optimum parameter.

### 5.5. Impact of Robust Scaler on the Performance of the Models

Just like the standard scaler, the regularized CNN outperformed all the other models when the robust scaler method was applied. The regularized CNN model has an accuracy of 95%, a precision score of 92%, a recall score of 92%, and an F1 score of 91%. It was followed by the CNN-BiLSTM which achieved an accuracy score of 86%, precision score of 83%, a recall score of 82%, and an F1-score of 74%. The KNN model achieved an accuracy score of 52%, a precision score of 91%, a recall score of 52%, and an F1-score of 66%, whereas the DT model achieved an accuracy score of 45%, a precision score of 43%, a recall score of 43%, and an F1-score of 43%. The results are presented below in Table 5.

**Figure 12:**
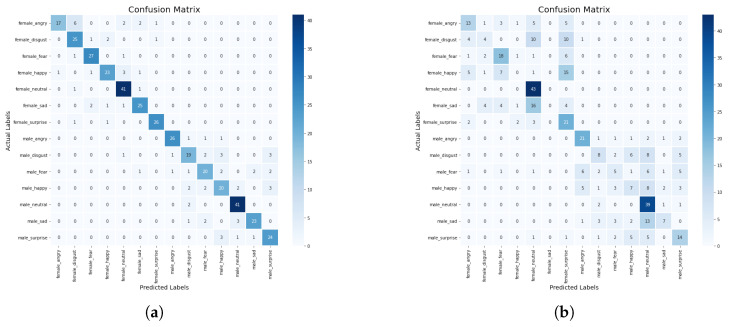
(**a**) Confusion matrix for CNN-BiLSTM model with robust scaler. (**b**) Confusion matrix for regularized CNN model with robust scaler.

**Figure 13:**
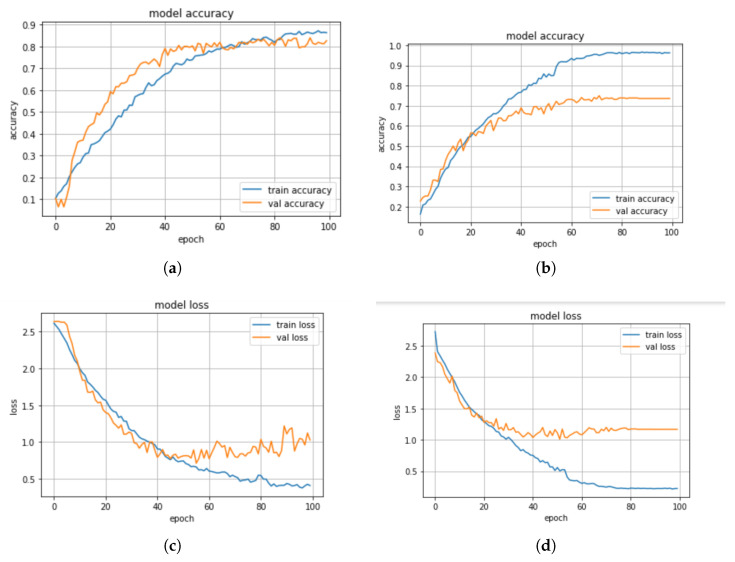
(**a**) Accuracy plot for the CNN-BiLSTM model with robust scaler. (**b**) Accuracy plot for the regularized CNN model with robust scaler. (**c**) Loss plot for the CNN-BiLSTM model with robust scaler. (**d**) Loss plot for the regularized CNN model with robust scaler.

The confusion matrix of the CNN-BiLSTM model and the regularized CNN model are presented in Figure 12a,b. Additionally, the accuracy and loss plots for the CNN-BiLSTM and the regularized CNN models are presented in Figure 13a–d, respectively. Furthermore, we compared the CNN-BiLSTM and the regularized CNN in terms of the difference between their actual values and their predicted values. For the CNN-BiLSTM model, the difference between the actual value and the predicted value is about 22%, while the regularized CNN model is about 10.5%. In this experiment, the two proposed models performed well. As a consequence, the performance improvement of the two models could be attributed to the optimization strategies that was employed to optimize the network parameters to find an optimum parameter. Moreover, to reduce the computational complexity in terms of computational time and space, we used techniques such as dropout, L1, and L2.

### 5.6. Performance Comparison of the Normalization Methods Based on the Proposed Deep Learning Models

**Figure 14:**
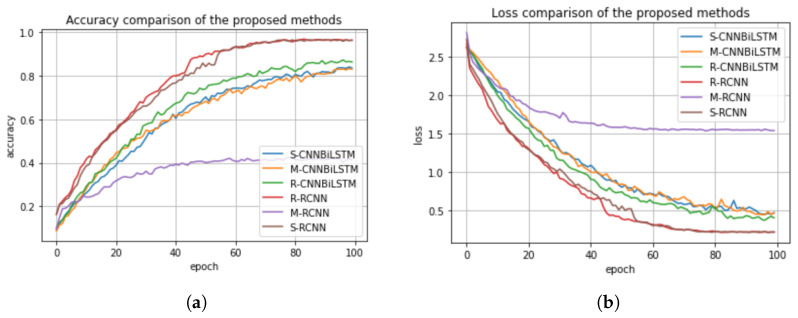
(**a**) Accuracy comparison of the normalization methods versus proposed models. (**b**) Loss comparison of the normalization methods versus proposed models.

In this section, we compare the normalization methods considered in this work, (i.e., standard scaler, min–max scaler, and the robust scaler) based on the classification performance of the proposed deep learning models. We present their accuracy and loss plots in Figure 14a,b. In Figure 14a,b, the standard, min–max, and robust scalers for the CNN-BiLSTM and the regularized CNN models are denoted as S-CNNBiLSTM, M-CNNBiLSTM, R-CNNBiLSTM and R-RCNN, M-RCNN, and S-RCNN, respectively. We can infer from the figures that the standard scaler and robust scaler performed well in the regularized CNN and in the CNN-BiLSTM, which achieved accuracy scores of 98% and 95%, respectively. Meanwhile, the min–max method performed poorly in the regularized CNN model which achieved an accuracy score of 47%, but then, it performed better in the CNN-BiLSTM with an accuracy score of 85%. Therefore, we conclude that the standard scaler has a great impact on the classification performance of the deep learning models.

## 6. Conclusions and Future Work

In this research work, we developed different machine and deep learning models to predict speech emotions in an IoT-enabled WBAN system. We described all the stages involved in the training and testing of the developed models, which covers different topics such as the data collection and review, data preparation, i.e., EDA and data processing, model building and evaluation. The dataset was reviewed based on the 5 V’s (i.e., volume, variety, velocity, veracity, value) of big data. For data preparation, we explored the dataset by visualizing it for better understanding and to check whether the dataset is balanced. For the data processing stage, we augmented the dataset, performed feature extraction, feature normalization, and addressed categorical data in the dataset. For the model building stage, we employed both supervised and unsupervised machine learning methods to construct the SER models. For instance, we developed a cluster method such as k-means with PCA, as well as developing different classification methods, such as KNN, DT, regularized CNN, and CNN-BiLSTM. We applied different feature normalisation methods on the developed models to investigate their impact on the classification performance.

For the purpose of evaluation and validation, the best models in our work (i.e., regularized CNN and CNN-BiLSTM) were compared with the most related state-of-the-art work in the literature using different performance metrics such as prediction accuracy, precision, recall, F1 score, confusion matrix, and differences between the actual and predicted values. From the results, it was observed that one of the proposed models (RCNN) outperformed the existing model. Additionally, the three normalization methods were compared in terms of accuracy and it was concluded that the standard scaler has a great impact on improving the accuracy of the models. In conclusion, future work linked to this study will focus on investigating more important speech features using deep learning algorithms. 

## Figures and Tables

**Table 1:** Parameter Settings.

Parameter	Value
Learning rate	0.001
Number of epochs	100
Batch size	64 units
Number of samples	1440
Data size	24.8 GB
Activation function	ReLU and Softmax
Optimization algorithm	Adam optimization
Hyperparameter optimization	Random search
Distance between audio device and access gateway	[0, 10] m
Regularization technique	L1, L2, dropout strategies

**Table 2:** A Comparison of the Proposed Model versus the Baseline Model.

Model	Accuracy	Precision	Recall	F1 Score
Regularized CNN	0.98	0.95	0.93	0.92
CNN-BiLSTM	0.85	0.82	0.80	0.81
Baseline model	0.95	0.92	0.92	0.90

**Table 3:** A Comparison of the Model Performance Based on Standard Scaler Method using Classification Report.

Model	Accuracy	Precision	Recall	F1 Score
KNN	0.52	0.91	0.52	0.64
DT	0.45	0.42	0.41	0.41
CNN-BiLSTM	0.85	0.82	0.80	0.81
Regularized CNN	0.98	0.95	0.93	0.92

**Table 4:** A Comparison of the Model Performance Based on Min–max Method using Classification Report.

Model	Accuracy	Precision	Recall	F1 Score
KNN	0.53	0.93	0.52	0.65
DT	0.44	0.42	0.41	0.40
CNN-BiLSTM	0.83	0.80	0.81	0.80
Regularized CNN	0.47	0. 40	0.68	0.68

**Table 5:** A Comparison of the Model Performance Based on Robust Scaler Method using Classification Report.

Model	Accuracy	Precision	Recall	F1 Score
KNN	0.52	0.91	0.52	0.66
DT	0.45	0.43	0.43	0.43
CNN-BiLSTM	0.86	0.83	0.82	0.74
Regularized CNN	0.95	0.92	0.92	0.92

## Data Availability

The dataset we used is available at https://pubmed.ncbi.nlm.nih.gov/29768426/ (accessed on 10 December 2022).
